# How Long May the Efficacy of Vaccination Be Relied on

**Published:** 1876-02

**Authors:** 


How Long may the Efficacy of Vaccination be Relied on.—Dr. Binkered (*Philadelphia Medical and Surgical Reporter*) says: "Jenner taught that protection by vaccination was life-long. It did not require the observations of half a century to disprove this statement of the great discoverer. The question 'How long?' can not with certainty be answered. It is generally agreed by all observing members of the profession that protection by thorough vaccination is for a few years certainly reliable. It is highly probable that one person may be protected by vaccination for a longer time than another person of equally distinct cicatrix. The range of susceptibility to any contagion is very wide in the human family. One individual will contract disease, while another equally exposed will escape with immunity. It is certainly safe to vaccinate indiscriminately every five years, or oftener if the disease prevails. At least make the effort. 'Best safety lies in fear.' But since specific disease enters so largely into the catalogue of human ills, we unhesitatingly commend the employment of animal vaccine virus. We have repeatedly vaccinated individuals well advanced in years and obtained a typical vesicle, though numerous efforts had failed in the same person in years gone by, subsequent to vaccination in early life."

